# Safety, efficacy, and clinical outcomes of transcatheter tricuspid valve replacement: One-year follow-up

**DOI:** 10.3389/fcvm.2022.1019813

**Published:** 2022-12-01

**Authors:** Yu Mao, Lanlan Li, Yang Liu, Mengen Zhai, Yanyan Ma, Chennian Xu, Ping Jin, Jian Yang

**Affiliations:** Department of Cardiovascular Surgery, Xijing Hospital, Air Force Medical University, Xi’an, China

**Keywords:** tricuspid regurgitation, transcatheter tricuspid valve replacement, LuX-Valve, follow-up, tricuspid valve

## Abstract

**Objective:**

The aim was to evaluate the safety and efficacy of TTVR in patients with severe TR at the 1-year follow-up.

**Materials and methods:**

This project was a single-center, observational study. From September 2020 to May 2021, 15 patients with severe or extremely severe TR at high risk of traditional surgery were enrolled. All patients had preoperative imaging assessments to evaluate the tricuspid valve and the anatomy of the right heart. All patients were planned to treated with the LuX-Valve (Ningbo Jenscare Biotechnology, Ningbo, China). The LuX-Valve was implanted under the intraoperative guidance of TEE and X-ray fluoroscopy. Data were collected at baseline, before discharge, and at 30 days, 6 months, and 1 year postoperatively.

**Results:**

The LuX-Valves were successfully implanted in all 15 patients. TR was significantly reduced to ≤ 2 +. One patient died on postoperative day 12 of a pulmonary infection that was considered unrelated to the procedures or the devices. The remaining 14 patients (100.0%) reached the primary end point. One patient (7.1%) was rehospitalized during 1-year follow-up because of device thrombosis. The number of patients who survived at 1 year with New York Heart Association (NYHA) functional class II was higher than that before TTVR (11/14 vs. 0/15, *P* = 9.11 × 10^–4^). Patients with peripheral edema and ascites decreased from 100.0 to 46.7% at baseline to 28.6% and 14.3% at 1 year (*P* = 1.57 × 10^–3^ and 2.53 × 10^–2^).

**Conclusion:**

TTVR is associated with RV remodeling, increased cardiac output, and improvement in NYHA functional class. Using the LuX-Valve for TTVR to treat patients with severe TR is a feasible and relatively safe method with reliable clinical results. Further studies are needed to determine long-term outcomes.

## Introduction

Tricuspid regurgitation (TR) is a common heart valve disease that is associated with increased mortality (1, 2). The prognosis of patients with severe TR is short of expectations, and the 5-year survival rate is less than 50% (2–5). TR is mainly secondary to dilation of the right ventricle (RV) and the tricuspid ring, which are closely associated with atrial fibrillation (AF) and pulmonary hypertension (6). The etiology of primary TR includes congenital tricuspid valve (TV) malformation, endocarditis, and a pacemaker implant. The traditional surgical treatment of TR involves TV repair and replacement assisted by a cardiopulmonary bypass device. Most patients with severe TR are treated with medication because interventions are associated with a high mortality rate, especially in the elderly (7–9). These results indicate that, for patients, annular repair may not be sufficient (10). Furthermore, the number of patients with TR is seriously underestimated, and less than 5% of patients receive surgical treatment (11).

In recent decades, transcatheter tricuspid valve replacement (TTVR) has become one of the research hotspots in cardiovascular medicine. Several interventional devices for different anatomical structures of the TV have been used clinically. Early reports from studies with these devices showed varying degrees of reduction of TR (12–20). The LuX-valve (Ningbo Jenscare Biotechnology, Ningbo, China) is one TTVR device unrelated to radial force that has been successfully implanted in patients with severe TR (21, 22). Our goal was to report the results of the 1-year follow-up in 15 patients with severe TR who received LuX-Valve implants.

## Materials and methods

### Study population

The study was a single-center, observational investigation. From September 2020 to May 2021, a total of 15 patients with severe TR [9 women; 62.0 (56.0, 78.0) years] were enrolled in this study. The severity of TR is classified as mild, moderate, severe, very severe, and extremely severe (23). All patients were carefully evaluated by the multidisciplinary cardiac team and considered to be either contraindicated or at high risk for surgery. According to European Society of Cardiology (ESC) and European Association of Cardiothoracic Surgery (EACTS) guidelines for the management of valvular heart disease, TR severity was graded as mild, moderate and severe in the present study evaluating by TR area (24). Meanwhile, TV is not a simple flat structure, but similar to the saddle oval. Therefore, in addition to assessing TR severity, the team also assessed the extent of TV annulus dilatation and cusp convolution (25). Inclusion criteria included age > 50 years old; TR severity ≥ severe; New York Heart Association (NYHA) class ≥ III; Patients at high risk for surgical tricuspid valve replacement as assessed by the multidisciplinary cardiac team [Society of Thoracic Surgeons (STS) score > 8.0%]. Exclusion criteria included left ventricular ejection fraction < 50%; systolic pulmonary arterial pressure > 55 mm Hg (1 mm Hg = 0.133 kPa); bioprosthetic valve replacement within 6 months; Ebstein’s malformation or structural dysplasia of the right ventricle; active infective endocarditis; cardiogenic shock; severe chronic renal insufficiency [glomerular filtration rate (GFR)< 30 mL/min]; combined with other heart disease requiring surgery. The clinical trial was registered in the ClinicalTrials.gov protocol registration system (NCT02917980). All procedures were in accordance with the ethical guidelines set out in the Declaration of Helsinki, and all patients signed the informed consent forms.

### Preoperative imaging

**FIGURE 1:**
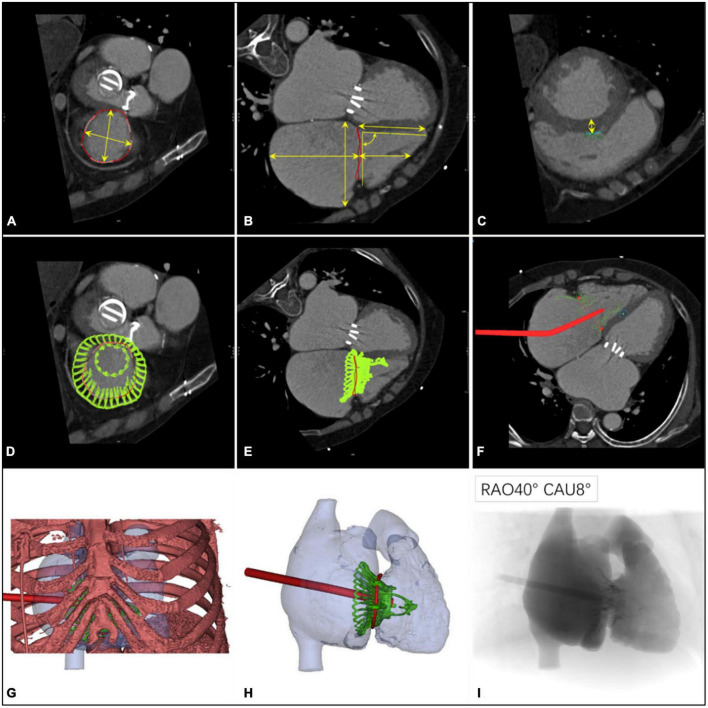
Preprocedural computerized tomography angiography assessment of transcatheter tri-cuspid valve replacement. **(A)** The diameter and perimeter of the tricuspid annulus (TA) were determined. **(B)** Measurements of the distance from the septal valve to the apex of the right ventricle, the height of the right atrium, and its relationship with the TA; the angle between the TA and the ventricular septum was 90° ± 10°. **(C–E)** Computer simulation of the LuX-Valve implant to observe the location of the anchor points and to measure the thickness of the ventricular septum in this position (30 mm below the TA). **(F)** Materialize Mimics 21.0 software (Materialize, Leuven, Belgium) was used to analyze the position and the angle of the delivery system on a 2-dimensional image. **(G)** The position of the right intercostal incision was determined with a digital 3-dimensional image. **(H)** The shape and the release position of the LuX-Valve were observed using 3-dimensional virtual models. **(I)** Simulation using fluoroscopic images provided an ideal projection angle for the transcatheter tricuspid valve replacement.

Coronary angiography was used to exclude severe coronary artery diseases; invasive RV catheterization was used to evaluate the hemodynamics of the right heart, and gated cardiac computed tomography and 3-dimensional reconstruction were used to evaluate anatomical structures. Functional TR is considered to be a disease that depends not only on the size and shape of the TV but also on the function of the RV, ventricular septal displacement, and pulmonary artery pressure (26). Transthoracic echocardiography (TTE) and transesophageal echocardiography (TEE) were both performed in all patients preoperatively to assess RV and TV functions (Figure 1).

### Device description

**FIGURE 2:**
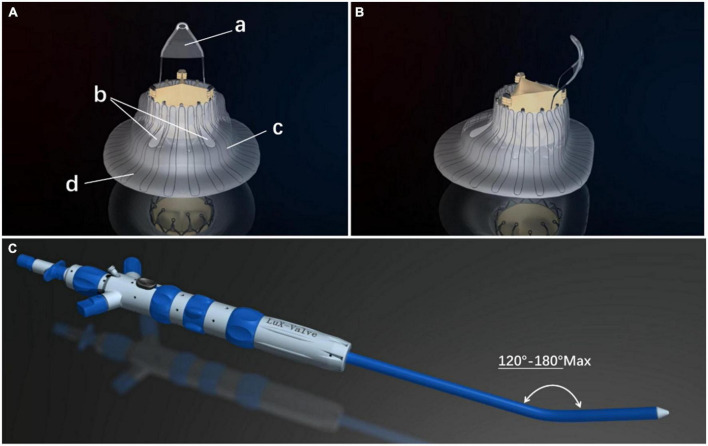
The LuX-Valve (Ningbo Jenscare Biotechnology, Ningbo, China) is self-expandable. The stent is made of a nickel–titanium alloy and the biological leaflet is bovine pericardium. The bio-prosthesis is implanted *via* the right atrial approach and fixed in the tricuspid annulus with its own unique anchoring device, independent of the radial support force. The part of the prosthesis located in the right atrium also prevents paravalvular leakage. **(A)** Right atrial view of the LuX-Valve. The four parts of the LuX-Valve stent include (a) the interventricular anchor, (b) two graspers, (c) the annulus skirt, and (d) the right atrial disc. **(B)** Lateral view of the LuX-Valve. **(C)** The delivery system of the LuX-Valve.

The LuX-Valve (Ningbo Jenscare Biotechnology, Ningbo, China) has a unique design concept of radial force independence, which consists of a biological valve stent, 3 valve lobules, and a steerable delivery system (Figure 2). It is funnel-shaped and consists of four parts: (a) A three-lobe artificial semilunar valve made of bovine pericardium treated with the GeniGal anticalcification process; (b) a self-expanding nitinol valve stent covered with polytetrafluoroethylene cloth, consisting of an atrial disc and soft adaptive annular sealing edges designed to prevent it from entering into the RV and to reduce paravalvular leakages; (c) the 20-mm “tongue” of the interventricular anchor (IVA), using a three-pronged nitinol anchor to grasp the valve stent to the diaphragm; (d) two 8-mm extended grips designed to capture the anterior TV ring. The delivery system consists of a 32 Fr sheath and a steerable tube. Four knobs, a plug, and a button on the handle control the bending of the sheath and the release of the valve.

### Procedural steps

**FIGURE 3:**
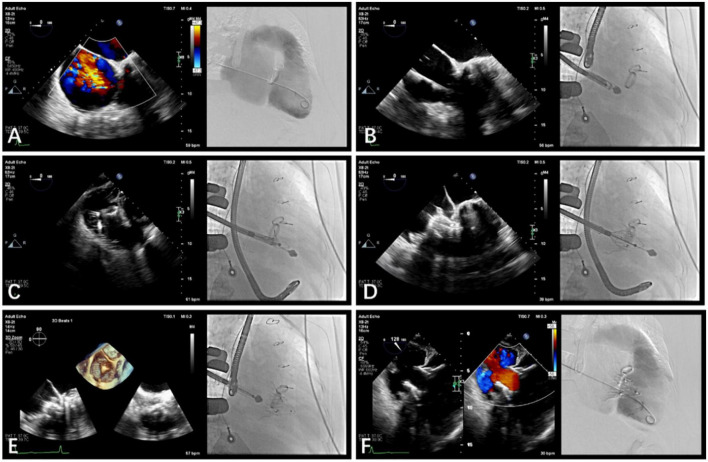
Guidance using transesophageal echocardiography (TEE) and fluoroscopic imaging in transcatheter tricuspid valve replacement. **(A)** TEE and fluoroscopy showed severe tricuspid re-gurgitation. **(B)** LuX-Valve guided by TEE was used to deliver the bioprosthesis to TA *via* the right intercostal approach. **(C)** The delivery system released the interventricular anchor and 2 graspers, and the graspers were guided by TEE to clamp the anterior leaflet. **(D)** The annulus skirt and the atrium disc were released in turn, and the position of the implant was adjusted by TEE to ensure that there was no obvious paravalvular leakage. **(E)** The bioprosthesis was completely re-leased after fixation with the interventricular anchor. **(F)** Postoperative computerized tomography angiography and TEE showed that tricuspid regurgitation disappeared immediately.

The procedure was performed in the intubation laboratory. After the patient was given general anesthesia, the TV was entered with a right minimally invasive thoracotomy through the path of the right atrium (RA) (Figures 3A,B). TEE and X-ray fluoroscopy were used for guidance. TEE was mainly used to guide catheter delivery, valve release, and adjustment of the intraoperative valve position. A coronary artery guide wire was placed in the right coronary artery to help determine the annulus plane of the TV. Systemic heparinization was administered to achieve an activated coagulation time of > 200 s; then 4-0 Prolene sutures with felt sheets were used with a double purse-string suture in the RA. The delivery catheter was placed into the RV under the guidance of TEE and X-ray fluoroscopy. The angle of the catheter was adjusted to ensure that the catheter was coaxial and centered with the ring. When the catheter was positioned under the loop, which was approximately 5 cm, the IVA and two clamping keys of the anterior lobes were released in turn by adjusting the knob system on the catheter (Figure 3C). Then, the clamping keys were positioned properly under the anterior lobe, and the entire delivery system was gently retracted so that the clamping keys hooked the anterior lobe. The atrial plate was released, the IVA was deployed, and the anchor pin was inserted into the septum for fixation (Figure 3D). Finally, the catheter was withdrawn and removed; then, the heparin was neutralized and the atrial incision was closed (Figures 3E,F).

### Data collection

Baseline data were collected from the electronic medical record system. The operative time, the device time, and the X-ray fluoroscopy time were recorded. The device time was defined as the time from catheter entry into the RA to withdrawal from the RA. In addition, data were collected during hospitalization (including the time in the intensive care unit and in the hospital and the postoperative TTE data).

### Follow-up

Follow-up data were collected from enrolled patients at baseline, before discharge, and at 30 days, 6 months, and 1 year postoperatively. Primary end points included a successful operation and a successfully implanted device. Successful surgery was defined as the successful implantation of the valve and removal of the delivery system; the correct and stable placement of the prosthesis; and no serious or life-threatening adverse events during the operation. The function of the TV was recovered satisfactorily [TR severity is reduced by ≥ 2, TV pressure gradient (PG) ≤ 6 mmHg], and there were no cardiovascular-related deaths, implant displacements, valve failures, or other major adverse events related to the device (including myocardial infarction, embolism, conduction disturbances, and a new transventricular septal shunt).

### Statistical analyses

Continuous variables were reported as the median (25th and 75th percentile), whereas classified variables were expressed by frequency and percentage. The paired *t*-test was used to compare continuous variables for each patient before and after the procedures, and other continuous variables were determined with the Student *t*-test. We compared the classification variables using the Wilcoxon signed rank test. A two-tailed *P*-value of < 0.05 was considered statistically significant. All statistical analyses were conducted using Statistical Package for Social Sciences (SPSS, Chicago, IL, USA) version 25.0.

## Results

### Baseline data

The baseline clinical features of the 15 patients are listed in Table 1. Despite receiving aggressive diuretic therapies, all patients had typical symptoms of severe right heart failure with ascites (46.7%) or peripheral edema (100.0%). In these 11 patients who had left-sided valvular surgery, 9 patients (81.8%) had been treated with surgical mitral valve replacement, and other 2 patients (18.2%) had been accepted with surgical mitral valve replacement and transcatheter aortic valve replacement. The causes of TR were left heart surgery (73.3%), permanent pacemaker or cardioverter defibrillator implants (40.0%), and AF (86.7%). Baseline echocardiographic and computed tomography (CT) parameters are listed in Table 2. All 15 patients had severe TR at baseline. Preoperative right heart catheterization showed that the systolic pulmonary arterial pressure of the included patients was 41.0 (32.0, 48.0) mm Hg, and 8 patients had pulmonary hypertension preoperatively. In addition, all patients were New York Heart Association (NYHA) functional class III/IV; the median European system for cardiac operative risk evaluation II was 9.5 (7.4, 11.6)% and the Society of Thoracic Surgeons score was 10.3 (7.8, 12.4)%, which indicated a high risk of cardiopulmonary bypass.

**TABLE 1:** Baseline patient characteristics (*N* = 15).

Characteristics	
Age (years)	62.0 (56.0, 78.0)
Female	9 (60.0)
Body mass index (kg/m^2^)	21.6 (19.1, 25.7)
NYHA class III or IV	15 (100.0)
STS score (%)	10.3 (8.2, 12.4)
EuroSCORE II (%)	9.5 (7.4, 11.6)
6MWT (m)	210.0 (155.0, 270.0)
KCCQ	32.0 (26.0, 39.0)
**Clinical symptoms**	
Peripheral edema	15 (100)
Ascites	7 (46.7)
**Blood sampling**	
Hemoglobin (g/L)	101.8 (91.4, 118.6)
Albumin (g/dL)	3.6 (3.2, 4.2)
Bilirubin (mg/dL)	1.2 (0.8, 1.5)
Creatinine (mg/dL)	1.0 (0.7, 1.3)
eGFR (mL/min)	56.7 (43.2, 69.8)
Troponin I (ng/mL)	3.9 (0.7, 11.7)
BNP (pg/mL)	202.1 (96.4, 353.9)
NT-proBNP (pg/mL)	775.0 (537.2, 1258.8)
Alanine transaminase (U/L)	16.3 (10.8, 25.6)
Aspartate transaminase (U/L)	28.0 (17.7, 41.0)
INR	1.5 (0.9, 2.1)
**Right heart catheterization**	
sPAP (mm Hg)	41.0 (32.0, 48.0)
mPAP (mm Hg)	24.0 (16.0, 32.0)
Pulmonary hypertension*	8 (53.3)
**Comorbidities**	
Diabetes	5 (33.3)
Atrial fibrillation	13 (86.7)
RBBB	3 (20.0)
LBBB	2 (13.3)
Coronary artery disease	2 (13.3)
Anemia	10 (66.7)
Dyslipidemia or hyperlipidemia	9 (60.0)
Chronic obstructive pulmonary disease	6 (40.0)
Chronic kidney disease†	7 (46.6)
Severe liver disease‡	5 (33.3)
Prior gastrointestinal hemorrhage	4 (26.6)
Prior stroke/TIA	1 (6.7)
**Previous cardiac intervention**	
Coronary artery bypass grafting	2 (13.3)
Left-sided valvular surgery	11 (73.3)
PPM/ICD	6 (40.0)

Values are presented as n (%) or median (25th, 75th percentile).

*mPAP ≥ 25 mm Hg.

†Defined as eGFR < 60 mL/min.

‡Defined as MELD-albumin score > 12. BNP, B-type natriuretic peptide; eGFR, estimated glomerular filtration rate; EuroSCORE, European system for cardiac operative risk evaluation; ICD, implantable cardioverter defibrillator; INR, international normalized ratio; KCCQ, Kansas City Cardiomyopathy Questionnaire; LBBB, left bundle branch block; MELD, Model for End-Stage Liver Disease; mPAP, mean pulmonary artery pressure; NT-proBNP, N-terminal pro-B-type natriuretic peptide; NYHA, New York Heart Association; PPM, permanent pacemaker; RBBB, right bundle branch block; 6MWT, 6-min walk test; sPAP, systolic pulmonary artery pressure; STS, Society of Thoracic Surgeons; TIA, transient ischemic attack.

**TABLE 2:** Baseline echocardiographic and computed tomography parameters (*N* = 15).

Echocardiographic parameters	
RV basal diameter (mm)	55.3 (43.5, 68.3)
RV mid diameter (mm)	42.0 (35.3, 50.5)
Fractional area change (%)	38.0 (32.7, 43.2)
TAPSE (mm)	13.0 (11.5, 16.0)
RV systolic TDI (cm/s)	10.0 (7.0, 14.0)
RA volume index (mL/m^2^)	88.0 (77.1, 121.2)
EROA PISA (mm^2^)	71.1 (62.0, 77.2)
LVIDD (mm)	40.0 (34.0, 55.0)
LVIDS (mm)	27.0 (21.0, 48.0)
LVEF (%)	54.0 (51.0, 65.0)
Transient regurgitation volume (mL)	72.6 (56.2, 110.3)
TR velocity (m/s)	2.86 (1.80, 3.67)
TA maximum diameters (mm)	48.4 (43.0, 52.1)
TA minimum diameters (mm)	40.5 (32.4, 47.0)
**Computed tomography parameters**	
TA maximum diameters (mm)	50.3 (44.8, 55.7)
TA minimum diameters (mm)	41.1 (36.1, 45.6)

EROA, effective regurgitation orifice area; LVEF, left ventricular ejection fraction; LVIDD, left ventricular internal dimension in diastole; LVIDS, left ventricular internal dimension in systole; PISA, proximal isovelocity surface area; RA, right atrium; RV, right ventricular; TA, tricuspid annular; TAPSE, tricuspid annular plane systolic excursion; TDI, tissue Doppler imaging; TR, tricuspid regurgitation.

### Intraoperative and hospitalization data

**FIGURE 4:**
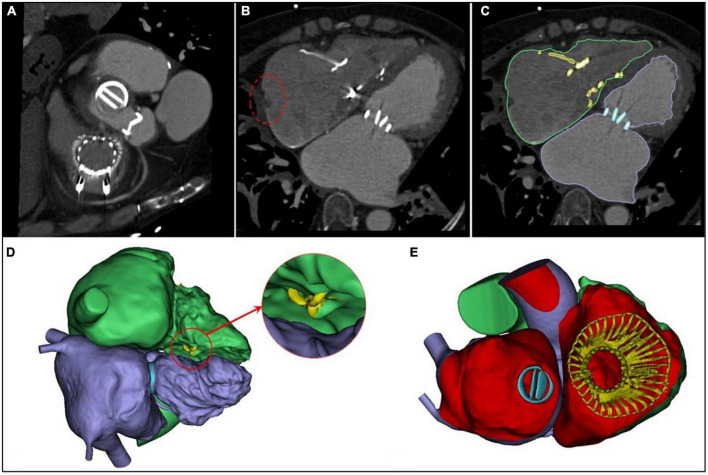
Postprocedural evaluation of the interventricular anchor. **(A)** A multislice computed tomography scan showed the precise positions of the two graspers. **(B)** The incision of the right atrium (the red circle) corresponds with Figure 1F. **(C)** The right heart is outlined in green; the left heart, in purple; the mechanical valve, in blue, and the LuX-Valve is yellow. **(D)** The yellow area in the red circle is the interventricular anchor. **(E)** The 3-dimensional reconstructed image from the right atrial plane demonstrates that the LuX-Valve is located in the normal position.

The intraoperative and hospitalization details are shown in Table 3. All patients were treated 3 to 5 days preoperatively and were given intravenous diuretics to reduce their weight and improve their peripheral edema. Surgical success was achieved in all patients (100%), with the individual valves in place in all cases. The operating time was 140.0 (110.0, 180.0) min, and the device time was 10.0 (7.0, 12.0) min, with no persistent ventricular arrhythmias, atrioventricular block, or cardiac rupture. In 6 patients who had previously been implanted with a permanent pacemaker or implantable cardioverter defibrillator, the lead remained attached to the RV with no change in threshold after the valve was implanted. After the procedures, TEE detected mild paravalvular leakage in 1 patient (6.6%), and moderate paravalvular leakage occurred in 1 patient (6.6%), possibly due to leaflet damage during the crimping of the valve. Postprocedural CT showed the precise location of the IVA and the two graspers (Figure 4). The remaining 13 patients (86.7%) had no/trace regurgitation. The mean postoperative times in the intensive care unit were 2.0 (1.0, 12.0) days, and the postoperative hospitalization times were 13.0 (7.0, 19.0) days. In patients with no preexisting renal impairment, RV angiography was performed to confirm the position and function of the implanted valve. Before discharge, CT was used to confirm the position and fixation details of the prosthesis. One patient died on postoperative day 12 of pulmonary infection, which was considered unrelated to the procedures or the devices. In addition, there were no pulmonary embolisms, cerebrovascular events, or new conduction blocks during hospitalization. All discharged patients were treated with anticoagulants. All patients had a ≥ 2 grade reduction in severity of TR from preoperative levels.

**TABLE 3:** Intraoperative and in-hospital outcomes (*N* = 15).

Intraoperative outcomes	
Procedural success	15 (100.0)
Procedural time (min)*	140.0 (110.0, 180.0)
Device time (min)†	18.0 (8.0,xf 26.0)
Fluoroscopy time (min)	23.0 (16.0, 31.0)
Bleeding volume (mL)	60.0 (30.0, 160.0)
**Intraoperative, postdevice TEE**	
Peak trans tricuspid gradient (mm Hg)	17.0 (8.0, 27.0)
Mean trans tricuspid gradient (mm Hg)	3.6 (1.8, 5.5)
Tricuspid valve area (cm^2^)	3.2 (2.1, 3.8)
**Complications**	
Conversion to median sternotomy	0 (0.0)
Right coronary injury	0 (0.0)
Perforation of right ventricle wall	0 (0.0)
New-onset conduction block	0 (0.0)
Atrioventricular block	0 (0.0)
Left bundle branch block	0 (0.0)
Right bundle branch block	0 (0.0)
**In-hospital outcomes**	
ICU length (days)	2.0 (1.0, 12.0)
Postoperative hospitalization length (days)	13.0 (7.0, 19.0)
Residual TR ≥ moderate‡	1 (6.6)
Postoperative 24-h chest drainage (mL)	170.0 (120.0, 875.0)
Myocardial infarction	0 (0.0)
Renal failure requiring dialysis	0 (0.0)
Gastrointestinal hemorrhage	0 (0.0)
Device migration	0 (0.0)
Device thrombosis	0 (0.0)
Pulmonary embolism	0 (0.0)
Pulmonary infection	1 (6.6)
Stroke/TIA	0 (0.0)
In-hospital deaths§	1 (6.6)
Troponin I (ng/mL)	0.16 (0.02, 0.30)
NT-proBNP (pg/mL)	689.3 (368.7, 1029.4)

Values are presented as n (%) or median (25th, 75th percentile).

*Defined as the duration from initial skin incision to final wound closure.

†Defined as the duration from guiding sheath insertion into the RA to retrieval of the delivery system.

‡One had central regurgitation and the others had perivalvular leakage. §One died during hospitalization of a lung infection. ICU, intensive care unit; TIA, transient ischemic attack; TR, tricuspid regurgitation.

### One-year follow-up data

**FIGURE 5:**
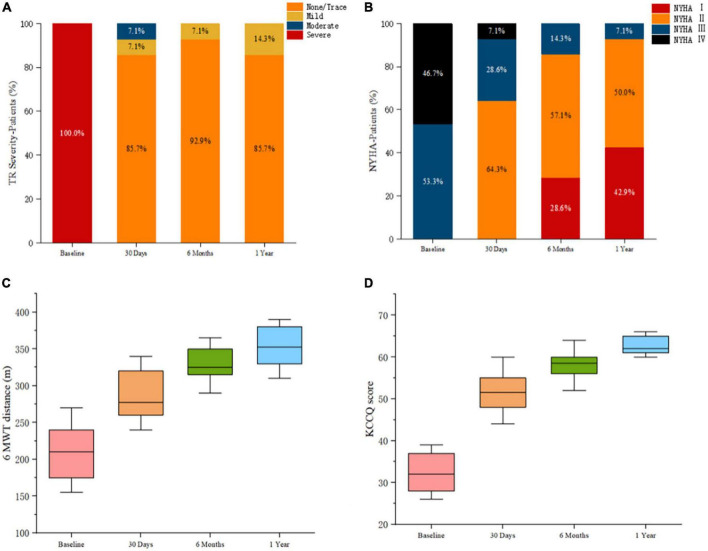
Postprocedural evaluation showed reduced severity of triscuspid regurgitation and improved clinical, functional, and quality-of-life outcomes. **(A)** Assessment of severity of tricuspid regurgitation. *P*-value calculated from the Wilcoxon signed rank test. **(B)** Comparison of New York Heart Association functional class pre-and post-procedures. *P*-value calculated from the Wilcoxon signed rank test. **(C)** Assessment of the 6-min walk test distances. *P*-value deter-mined from the paired Student *t*-test. **(D)** Assessment using the Kansas City Cardiomyopathy Questionnaire. *P*-value determined from the paired Student *t*-test.

Major follow-up outcomes at 1 year are shown in Table 4. Baseline to 1-year echocardiographic measurements are listed in Table 5. For 14 patients, TR severity measured by TTE decreased from 100.0% severe to 85.7% no/trace (*P* = 5.32 × 10^–4^). Of the remaining patients, 1 patient had mild paravalvular leakage, and another patient had moderate paravalvular leakage. TA diameter and RV diameter were both decreased compared with preoperative measurements, indicating RV remodeling. All patients exhibited significant improvement in symptoms at 6 months. For the 6-month follow-up data, the TR decreased to no/trace in 13 patients (92.9%, *P* = 3.11 × 10^–4^). One patient had mild paravalvular leakage. At the 1-year follow-up, TR decreased to no/trace in 12 patients (85.7%, *P* = 5.32 × 10^–4^). Two patients had mild paravalvular leakage. In addition, the reduction of the TV ring diameter and the increased deviation of the TV annular plane in systole indicated improvement in RV structure and function. Meanwhile, the TAPSE measurement improved significantly [16.3 (14.4, 18.8) vs. 13.0 (11.5, 16.0), *P* = 3.63 × 10^–5^], and the RV volume showed remarkable improvement [59.3 (47.5, 68.5) vs. 80.5 (66.0, 96.5), *P* = 1.06 × 10^–11^]. Furthermore, peripheral edema and ascites decreased to 28.6 and 14.3%, respectively (*P* = 1.57 × 10^–3^ and 2.53 × 10^–2^). The proportion of patients in NYHA functional class II was higher than that before the operation (11/14 vs. 0/15, *P* = 9.11 × 10^–4^). The 6-min walking test results showed significant improvement in motion performance [355.0 (310.0, 390.0) m vs. 210.0 (155.0, 270.0) m, *P* = 9.56 × 10^–14^). Kansas City cardiomyopathy questionnaire scores also improved significantly at the 1-year follow-up [62.0 (60.0, 66.0) vs. 32.0 (26.0, 39.0), *P* = 9.29 × 10^–15^]. Thirteen patients (92.9%) met the primary end points. One patient (7.1%) was re-hospitalized because of device thrombosis (Figure 5). Due to the LuX-Valve has a larger atrial plate compared to other devices, the bioprosthetic valve effectively prevents paravalvular leakage but is apt to thrombose. Furthermore, the lower pressure of the RV results in slower blood flow in comparison to blood flow through the left ventricle, and the dosage of anticoagulation has not been determined in the current studies.

**TABLE 4:** Follow-up outcomes at 1 year after discharge (*N* = 14).

1-year deaths	0 (0.0)
Myocardial infarction	0 (0.0)
Rehospitalization*	1 (7.1)
Renal complications requiring dialysis	0 (0.0)
Need for renal replacement therapy	0 (0.0)
Non-elective tricuspid valve reintervention	0 (0.0)
Device migration	0 (0.0)
Device thrombosis	1 (7.1)
Severe bleeding	0 (0.0)
Major cardiac structural complications	0 (0.0)
Pulmonary embolism	0 (0.0)
Gastrointestinal hemorrhage	0 (0.0)
Stroke/TIA	0 (0.0)
New-onset third-degree atrioventricular block	0 (0.0)

Values are presented as n (%).

*One patient was rehospitalized due to device thrombosis.

TIA, transient ischemic attack.

**TABLE 5:** Baseline to 1-year echocardiographic measurements.

Echocardiographic parameters	Baseline (*N* = 15)	30 days (*N* = 14)		6 months (*N* = 14)		1 year (*N* = 14)	
		Results	*P* value	Results	*P* value	Results	*P* value
**TR severity**							
None/trace	0 (0.0)	12 (85.7)	5.32 × 10^–4^	13 (92.9)	3.11 × 10^–4^	12 (85.7)	5.32 × 10^–4^
Mild	0 (0.0)	1 (7.1)	0.32	1 (7.1)	0.32	2 (14.3)	0.16
Moderate	0 (0.0)	1 (7.1)	0.32	0 (0.0)	—	0 (0.0)	—
Severe	15 (100.0)	0 (0.0)	1.08 × 10^–4^	0 (0.0)	1.08 × 10^–4^	0 (0.0)	1.08 × 10^–4^
TAPSE (mm)	13.0 (11.5, 16.0)	13.9 (12.4, 16.5)	6.31 × 10^–4^	15.7 (13.6, 18.0)	6.48 × 10^–5^	16.3 (14.4, 18.8)	3.63 × 10^–5^
Fractional area change (%)	38.0 (32.7, 43.2)	39.6 (34.3, 46.4)	2.87 × 10^–8^	40.8 (35.2, 47.5)	3.10 × 10^–9^	41.3 (35.7, 47.8)	3.75 × 10^–11^
EROA PISA (mm^2^)	71.1 (62.0, 77.2)	—	—	—	—	—	—
Peak transtricuspid gradient (mm Hg)	18.5 (8.0, 32.0)	6.5 (4.0, 11.0)	5.73 × 10^–15^	5.0 (3.0, 8.0)	3.82 × 10^–15^	5.5 (3.0, 13.0)	4.33 × 10^–15^
Mean transtricuspid gradient (mm Hg)	2.0 (1.3, 3.3)	3.5 (2.4, 4.5)	5.92 × 10^–14^	2.6 (1.8, 3.6)	7.91 × 10^–10^	2.3 (1.4, 3.0)	4.04 × 10^–3^
RV basal diameter (mm)	55.3 (43.5, 68.3)	52.5 (41.7, 62.3)	9.62 × 10^–4^	49.8 (41.4, 58.5)	6.96 × 10^–4^	48.9 (40.5, 56.6)	6.80 × 10^–5^
RV mid diameter (mm)	42.0 (35.3, 50.5)	37.7 (32.1 46.4)	4.52 × 10^–12^	36.0 (31.6, 44.0)	5.58 × 10^–13^	35.2 (30.8, 43.3)	2.48 × 10^–13^
RV volume (mL)	80.5 (66.0, 96.5)	68.3 (54.8, 77.0)	1.59 × 10^–11^	63.0 (50.5, 73.8)	1.37 × 10^–11^	59.3 (47.5, 68.5)	1.06 × 10^–11^
RA volume (mL)	188.0 (134.5, 253.0)	159.8 (120.3, 220.0)	5.69 × 10^–8^	142.0 (112.8, 206.3)	2.55 × 10^–9^	131.5 (104.5, 201.0)	7.33 × 10^–10^

Values are presented as N (%) or median (25th, 75th percentile).

EROA, effective regurgitation orifice area; LA, left atrium; LV, left ventricle; LVEF, left ventricular ejection fraction; PISA, proximal isovelocity surface area; RA, right atrium; RV, right ventricle; TAPSE, tricuspid annular plane systolic excursion; TR, tricuspid regurgitation.

## Discussion

In this single-center, observational study, the LuX-Valve was successfully implanted in all 15 patients, and good clinical results were achieved without the complex TV anatomical structures and different etiologies. The unique anatomical structures and pathophysiological characteristics of the TV make the TTVR device difficult to design. From a physiological point of view of, the TV has a 3-dimensional structure similar to that of a saddle that exhibits dynamic changes during the cardiac cycle to ensure that the valve closes completely. Primary TR is caused by congenital or acquired abnormalities of the TV itself. However, secondary (or functional) TR, which is far more common than primary TR, is secondary to excess RV pressure and/or volume load. When TR occurs, the TV loses its normal shape and dilates under the strain of the dilated RA and RV. Recent studies suggest that the overloading of the RV caused by long-term TR may lead to irreversible myocardial injury of the RV (27). As a result, as the focus on TR has increased, the number of operations on the TV has increased (27). Most studies have reported incomplete reduction of TR (14, 28, 29). A recent large registry of patients who had transcatheter aortic valve replacement showed that the severity of preoperative TR was independently associated with 1-year postoperative mortality and rehospitalization for heart failure (30). In general, the TV may not provide stable support for traditional radial TTVR devices. The LuX-Valve is an *in situ* TTVR device with a non-radial support force that has unique advantages compared with those of the traditional radial support force devices. The selection of the valve size is based on the effective orifice area rather than on the expanded TV, which renders the selection of diameter sizes smaller. This design also ensures that the diameter of the annulus decreases as the RV remodeling reverses. In addition, the smaller valve has no radial support on the TV, so it is almost impossible to induce right ventricular outflow tract (RVOT) obstruction, right coronary artery injury, or conduction block (13). The LuX-Valve has a larger atrial plate compared to other devices, which effectively prevents paravalvular leakage after the valve is implanted. These advantages suggest that the LuX-Valve is suitable for the treatment of TR caused by a variety of etiologies, including functional TR, TR caused by the pacemaker lead, and chronic AF. Hahn reported that NaviGate system (NaviGate Cardiac Structures, Lake Forest, CA, USA), which was a radial force-dependent TTVR device. However, the patients who received NaviGate implantation had a high prevalence of bioprosthesis failure, atrioventricular block and paravalvular leakage (31). During the 1-year follow-up of this small series of patients with severe, symptomatic TR treated with TTVR, there were a number of important observations. First, TTVR virtually eliminates TR or underlying disease. Despite multiple comorbidities, those who survived to 1 year had RV remodeling and increased cardiac output. Previous studies have shown that changes of RV dimensions and function would predict TR after TTVR. RV systolic function is mainly determined by afterload, preload, and intrinsic myocardial contractility (32). With the significant decrease in TR after procedures, an increase in afterload may affect RV function or even induce irreversible changes. However, the further studies are needed to proceed. Second, successful procedures depend on the guidance of TEE and CTA. Preimplantation sizing may be adjusted in a number of different ways. In fact, even advanced 3-dimensional reconstruction tools are used. Third, due to the lack of obvious anatomical markers of TV under the guidance of digital subtraction angiography, accurate positioning is required when the LuX-Valve is implanted. Fourth, the increased incidence of pulmonary complications caused by bleeding in the chest should be prevented during the procedures. Fifth, the lower pressure of the RV results in slower blood flow in comparison to blood flow through the left ventricle, so anticoagulation is needed to prevent valve thrombosis. However, further research is needed to determine whether vitamin K antagonists, direct oral anticoagulants, or dual antiplatelet agents should be used.

At present, the morbidity of patients with severe TR is high, but the treatment effect is not satisfied, so the market prospect of interventions for TR in the future is broad. However, not all patients with TR meet the indications for interventions. In addition, many patients present with right heart failure and other manifestations at the time, so the perioperative management of patients with TR is more challenging. When selecting patients in the future, it is necessary to strengthen the evaluation of anatomical characteristics and comorbidities of the specific patient at the same time, and continuously improve the quality of surgical and perioperative management.

### Limitations

This study has some limitations. First, this study lacks a control group undergoing traditional surgery (Such as a propensity score matched control group of patients with surgical tricuspid valve replacement *via* right thoracotomy), which requires a larger sample size and a well-designed clinical trial to confirm its long-term safety and effectiveness. Second, the use of the LuX-Valve is limited because the surgical approach is still through a thoracic incision, and its delivery system needs to be further improved to be implanted through the peripheral vein path. Third, whereas an average of multiple cardiac cycles is used to measure most RV parameters, strain imaging uses a single cycle and may not represent the entire RV function for patients with AF. Finally, the follow-up time was limited.

## Conclusion

The patients with severe functional TR were treated by TTVR, which is a feasible, relatively safe and low-complication approach that improves RV remodeling and relieves symptoms of right heart failure with reliable clinical outcomes.

## Data availability statement

The original contributions presented in this study are included in the article/supplementary material, further inquiries can be directed to the corresponding author.

## Ethics statement

The studies involving human participants were reviewed and approved by the Xijing Hospital Ethics Committee, ClinicalTrials.gov protocol registration system (NCT02917980). The patients/participants provided their written informed consent to participate in this study. Written informed consent was obtained from the individual(s) for the publication of any potentially identifiable images or data included in this article.

## Author contributions

YuM, LL, and YL were responsible for wrote the manuscript. MZ and YaM were responsible for the figures. CX and PJ were responsible for the data collecting. JY was responsible for the manuscript reviewing. All authors contributed to the article and approved the submitted version.
